# Supplementary material for: Effectiveness and Safety of Ayurvedic Medicines in Type 2 Diabetes Mellitus Management: A Systematic Review and Meta-Analysis
Source: Front Pharmacol. 2022 Jun 8;13:821810. doi: 10.3389/fphar.2022.821810 (PMC9213670; doi:10.3389/fphar.2022.821810)
Supplement: Supplementary file 1 [file DataSheet1.zip › Supplementary Material/Forest and Funnel Plots/Trigonella foenum-graecum/Body weight.pdf]

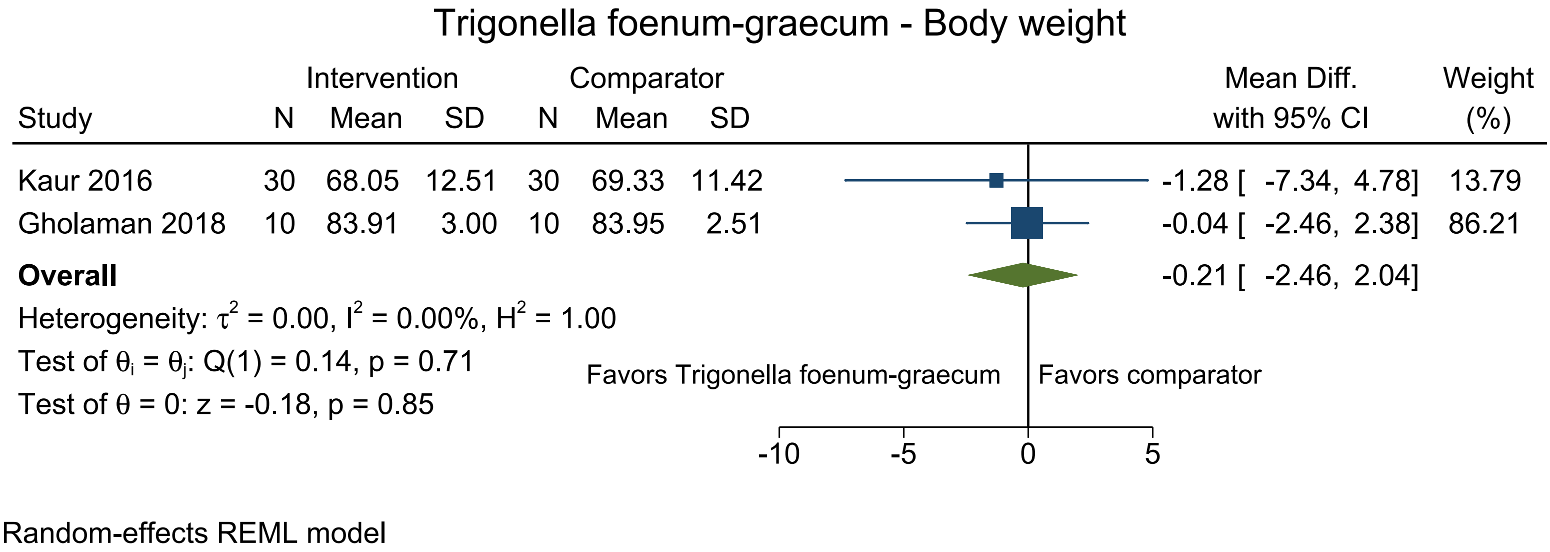
Trigonella foenum-graecum - Body weight
Intervention
Comparator
Mean Diff. with 95% CI
Weight (%)
Study N Mean SD N Mean SD
Kaur 2016 30 68.05 12.51 30 69.33 11.42 -1.28 [-7.34, 4.78] 13.79
Gholaman 2018 10 83.91 3.00 10 83.95 2.51 -0.04 [-2.46, 2.38] 86.21
Overall -0.21 [-2.46, 2.04]
Heterogeneity: τ² = 0.00, I² = 0.00%, H² = 1.00
Test of θi = θj: Q(1) = 0.14, p = 0.71
Test of θ = 0: z = -0.18, p = 0.85
Favors Trigonella foenum-graecum
Favors comparator
-10
-5
0
5
Random-effects REML model
